# Vessel and sex differences in pericoronary adipose tissue attenuation obtained with coronary CT in individuals without coronary atherosclerosis

**DOI:** 10.1007/s10554-022-02716-7

**Published:** 2022-08-24

**Authors:** Sophie E. van Rosendael, Jurrien H. Kuneman, Inge J. van den Hoogen, Pieter H. Kitslaar, Alexander R. van Rosendael, Pieter van der Bijl, Johan H. C. Reiber, Nina Ajmone Marsan, J. Wouter Jukema, Juhani Knuuti, Jeroen J. Bax

**Affiliations:** 1 Department of Cardiology, Leiden University Medical Centre, Albinusdreef 2, 2333ZA Leiden, The Netherlands; 2 Medis Medical Imaging Systems, Leiden, The Netherlands; 3 Division of Image Processing, Department of Radiology, Leiden University Medical Centre, Leiden, The Netherlands; 4 Netherlands Heart Institute, Utrecht, The Netherlands; 5 Turku PET Centre, Turku University Hospital and University of Turku, Turku, Finland; 6 Heart Centre, University of Turku and Turku University Hospital, Turku, Finland

**Keywords:** Coronary artery disease, Coronary computed tomography angiography, Pericoronary adipose tissue attenuation, Perivascular inflammation

## Abstract

Pericoronary adipose tissue (PCAT) attenuation, derived from coronary computed tomography angiography (CCTA), is associated with coronary artery inflammation. Values for PCAT attenuation in men and women without atherosclerosis on CCTA are lacking. The aim of the current study was to assess the mean PCAT attenuation in individuals without coronary artery atherosclerosis on CCTA. Data on PCAT attenuation in men and women without coronary artery atherosclerosis on CCTA were included in this retrospective analysis. The PCAT attenuation was analyzed from the proximal part of the right coronary artery (RCA), the left anterior descending artery (LAD), and the left circumflex artery (LCx). For patient level analyses the mean PCAT attenuation was defined as the mean of the three coronary arteries. In 109 individuals (mean age 45 ± 13 years; 44% men), 320 coronary arteries were analyzed. The mean PCAT attenuation of the overall population was − 64.4 ± 8.0 HU. The mean PCAT attenuation was significantly lower in the LAD compared with the LCx and RCA (− 67.8 ± 7.8 HU vs − 62.6 ± 6.8 HU vs − 63.6 ± 7.9 HU, respectively, p < 0.001). In addition, the mean PCAT attenuation was significantly higher in men vs. women in all three coronary arteries (LAD: − 65.7 ± 7.6 HU vs − 69.4 ± 7.6 HU, p = 0.014; LCx: − 60.6 ± 7.4 HU vs − 64.3 ± 5.9 HU, p = 0.008; RCA: -61.7 ± 7.9 HU vs − 65.0 ± 7.7 HU, p = 0.029, respectively). The current study provides mean PCAT attenuation values, derived from individuals without CAD. Moreover, the mean PCAT attenuation is lower in women vs. men. Furthermore, the mean PCAT attenuation is significantly lower in the LAD vs LCx and RCA.

**Supplementary Information:**

The online version contains supplementary material available at 10.1007/s10554-022-02716-7.

## Introduction

Vascular inflammation contributes to coronary atherosclerotic plaque formation and atherosclerotic plaque rupture [1–3]. Over the past years, the link between pericoronary adipose tissue (PCAT) associated inflammation and atherosclerosis has been demonstrated in several studies [4–6]. PCAT attenuation reflects vascular inflammation, which is associated with unstable plaque features and is considered a sensitive inflammatory biomarker which may improve cardiovascular risk stratification [4, 7]. Vascular inflammation can influence adipocyte lipid content through paracrine signalling by affecting biological processes such as adipocyte differentiation, proliferation and lipolysis in adjacent perivascular fat [4, 8]. Moreover, Antonopoulos et al. [4] demonstrated an inverse association of PCAT attenuation on coronary computed tomography angiography (CCTA) with histological adipocyte size and degree of adipocyte differentiation. Moreover, the authors demonstrated that PCAT with higher attenuation values on CCTA was correlated with smaller adipocytes with lower lipid content [4]. Many previous studies assessed PCAT attenuation in patients with atherosclerotic coronary arteries, whereas PCAT attenuation values in individuals without coronary atherosclerosis are lacking. Accordingly, the purpose of the current study is to evaluate the PCAT attenuation in the right coronary artery (RCA), the left anterior descending artery (LAD) and the left circumflex artery (LCx) in individuals without coronary atherosclerosis, to establish reference values.

## Methods

### Study design and participants

**Fig. 1:**
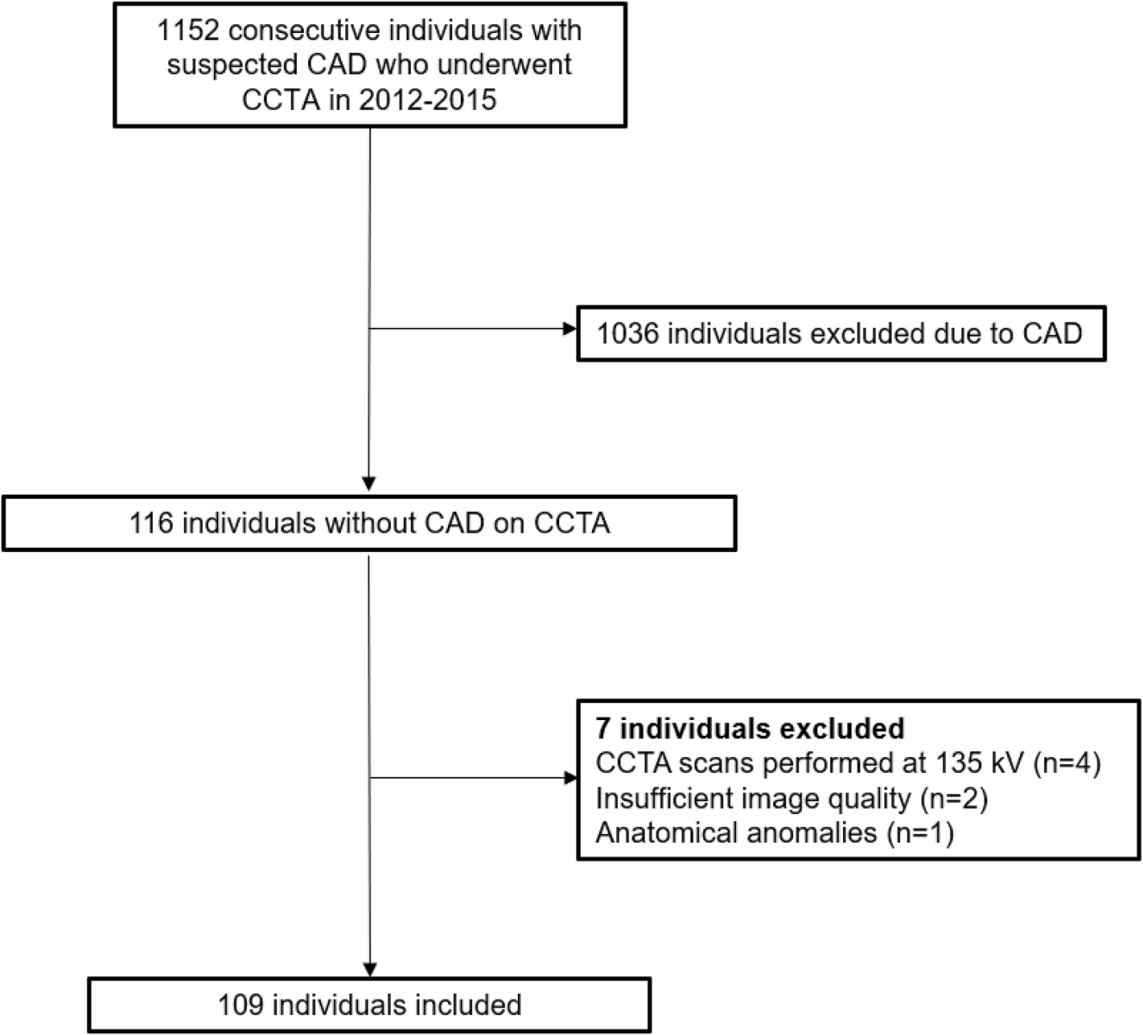
Study flowchart, *CAD* coronary artery disease, *kV* kilovoltage, *CCTA* coronary computed tomography angiography

Consecutive individuals without coronary atherosclerosis on CCTA at the Leiden University Medical Centre (Leiden, The Netherlands) between 2012 and 2015 were identified and included in this retrospective, observational analysis. Individuals with suboptimal CCTA image quality or coronary anomalies, as well as individuals who had a CCTA scan at a tube voltage of 135 kV, were excluded (Fig. 1). Baseline clinical demographic characteristics including medication use and cardiovascular risk factors were reported. The current study was conducted in accordance with the Declaration of Helsinki. The study protocol was approved by the local ethics committee, who waived the need for written informed consent.

### CCTA image acquisition

All CCTA scans were performed with a 320-slice multi-detector computed tomography scanner (Aquilion ONE, Toshiba Medical Systems, Otawara, Japan) with a gantry rotation time of 350 ms. Tube voltage and tube current varied from 100–120 kV to 150–640 mA, depending on the individual’s size. If the heart rate before the CCTA scan was > 65 beats per minute, 25–150 mg of oral metoprolol was administered 1 h before the CCTA scan, unless contraindicated. If the heart rate remained > 65 beats per minute during the CCTA scan, up to 10 mg of intravenous metoprolol was administered additionally. Sublingual nitroglycerin (400–800 μg) was administered to all individuals before the scan.

### CCTA analysis

Anatomical CCTA evaluation was performed using the 17-segment modified American Heart Association model [9]. Quantitative CCTA analysis was performed using dedicated software (QAngio CT Research Edition version 3.2.0.13; Medis Medical Imaging Systems, Leiden, The Netherlands). In brief, a 3-dimensional coronary tree was derived from the CCTA images. All coronary arteries with a diameter of ≥ 1.5 mm were evaluated for the presence of atherosclerosis. For each coronary artery, multiplanar reconstructions were created. Lumen and vessel wall contours were automatically detected, with manual correction of the lumen vessel contours if needed [10]. The presence of coronary atherosclerosis was defined as a tissue structure > 1 mm [2] within or adjacent to the coronary artery lumen that could be distinguished from surrounding pericardial tissue, epicardial fat, or the vessel lumen itself [11].

### Pericoronary adipose tissue attenuation analysis

**Fig. 2:**
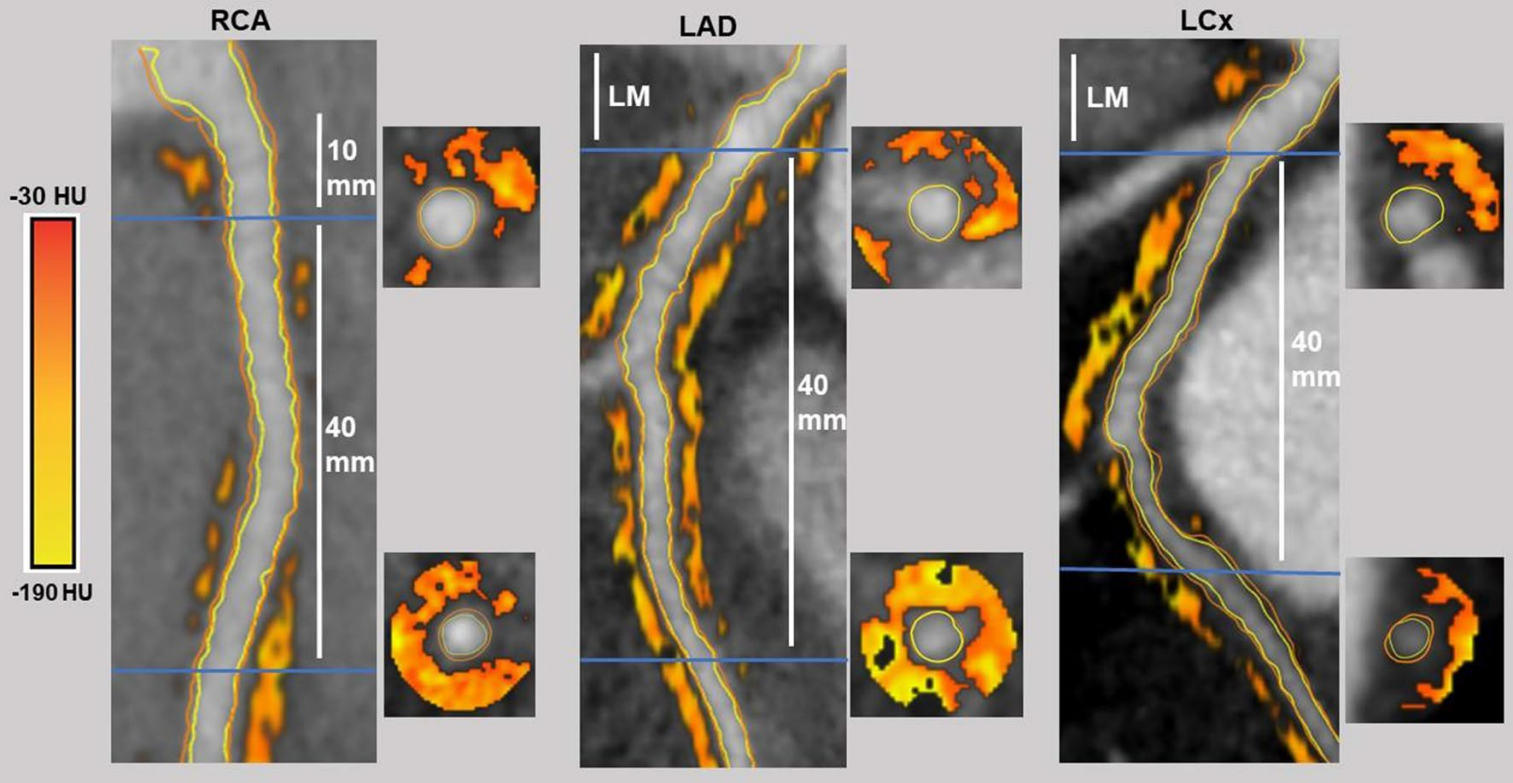
Pericoronary Adipose Tissue (PCAT) analysis of the proximal segments of the left anterior descending artery (LAD), the left circumflex artery (LCx) and the right coronary artery (RCA). Including corresponding pericoronary adipose tissue colour maps and cross-sectional views of the start and end point of the analyzed segments, *HU* Hounsfield unit

The mean pericoronary adipose tissue (PCAT) attenuation was evaluated in all three major epicardial coronary arteries using dedicated software (QAngio CT Research Edition version 3.2.0.13, Medis Medical Imaging Systems, Leiden, The Netherlands). The PCAT was defined as the area with an attenuation between − 30 and − 190 Hounsfield Units (HU) within a radial distance from the outer vessel wall equivalent to the diameter of the vessel [4, 7]. The proximal 40 mm segments of the LAD and LCx were analyzed (Fig. 2). The proximal 10–50 mm segment of the RCA was evaluated, in order to avoid effects of the aortic wall (Fig. 2) [4]. To adjust for differences in attenuation between scans performed at different tube voltages, the mean PCAT attenuation of CCTA scans performed at 100 kV was divided by a conversion factor of 1.11485 [4, 12]. At a per-patient level, the mean PCAT attenuation was defined as the average of the three major coronary arteries. A minimal artery length of 40 mm was necessary for PCAT attenuation measurements.

### Statistical analysis

SPSS version 25 (IBM SPSS Statistics, IBM Corporation, Armonk, New York, USA) was used for statistical analyses. Continuous variables with a normal distribution are presented as mean ± standard deviation and were compared using the Student t-test or the one-way ANOVA test, as appropriate. The Bonferroni correction was applied in case of a significant difference in the overall three group comparison. Distribution of continuous variables was evaluated using histograms. Categorical variables are presented as absolute numbers and percentages and were compared using the χ^2^ test. The correlation of the mean PCAT attenuation between the various epicardial coronary arteries was evaluated using the Pearson correlation test. Linear regression analyses were performed to investigate the association between mean PCAT attenuation and sex, adjusted for smoking status. A two-sided p-value < 0.05 was considered significant.

## Results

### Baseline characteristics

In total, 109 individuals (mean age 45 ± 13 years; 44% male) including 320 coronary arteries without atherosclerosis on CCTA were included. A flowchart of the population is displayed in Fig. 1. Seven coronary arteries (LCx: n = 5, RCA: n = 2) were too small for PCAT attenuation analysis and were excluded. Baseline demographic and clinical characteristics of the overall population and according to sex are shown in Table 1. Of the overall population, hypertension was present in 28 (25.7%) and dyslipidemia in 13 individuals (11.9%). Men were more often smokers as compared to women (27.1 vs 11.5%, p = 0.037).

**Table 1:** Baseline characteristics of the population and according to sex

	Overall population (n = 109)	Men (n = 48)	Women (n = 61)	p-value
Age, years	45 ± 13	44 ± 13	46 ± 13	0.426
BMI, kg/m^2^	24.6 ± 3.8	24.6 ± 3.6	24.7 ± 3.9	0.850
Symptoms, n (%)				
Typical angina	5 (4.6)	2 (4.2)	3 (4.9)	0.852
Atypical angina	39 (35.8)	14 (29.2)	25 (41.0)	0.201
Non-anginal	21 (19.2)	9 (18.8)	12 (19.7)	0.904
No pain	44 (40.4)	23 (47.9)	21 (34.4)	0.154
Dyspnea	7 (6.4)	2 (4.2)	5 (8.2)	0.394
Cardiac risk factors, n (%)				
Hypertension	28 (25.7)	8 (16.7)	20 (32.8)	0.056
Dyslipidemia	13 (11.9)	6 (12.5)	7 (11.5)	0.870
Diabetes mellitus	12 (11)	5 (10.4)	7 (11.5)	0.861
Family history of CAD	46 (42.4)	23 (47.9)	23 (37.7)	0.284
Current smoking	20 (18.3)	13 (27.1)	7 (11.5)	0.037
Obesity	12 (11.1)	3 (6.4)	9 (14.8)	0.150
Cardiovascular medication, n (%)				
Aspirin	15 (13.8)	9 (9.5)	24 (18.9)	0.801
Beta-blockers	6 (5.4)	10 (20.8)	18 (29.5)	0.333
Calcium channel blockers	28 (25.7)	1 (2.1)	4 (6.6)	0.277
ACE-inhibitors or angiotensin II receptor blockers	5 (4.6)	4 (8.3)	10 (16.4)	0.227
Diuretics	6 (5.4)	0 (0)	6 (9.8)	0.027
Statins	16 (14.7)	5 (10.4)	11 (18)	0.284
Tube voltage, n (%)				
100 kV	80 (74.1)	35 (74.4)	45 (73.8)	0.935
120 kV	28 (25.9)	12 (25.5)	16 (26.2)	0.935

Data are presented as mean ± SD and n (%) *BMI* body mass index, *CAD* coronary artery disease, *kV* kilovoltage

### Pericoronary adipose tissue attenuation

**Fig. 3:**
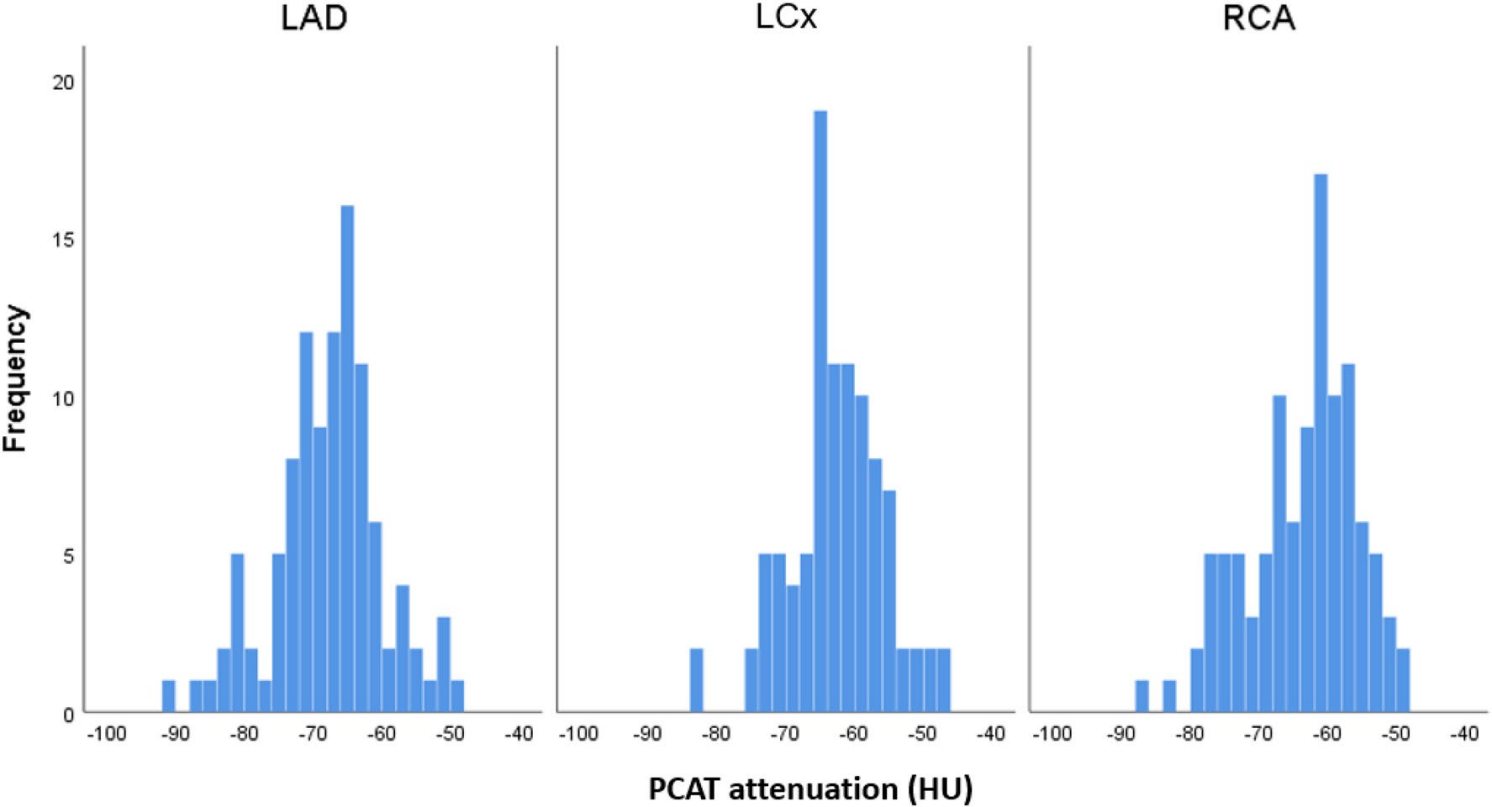
Distribution of the mean pericoronary adipose tissue (PCAT) attenuation of the left anterior descending artery (LAD), the left circumflex artery (LCx) and the right coronary artery (RCA)

**Fig. 4:**
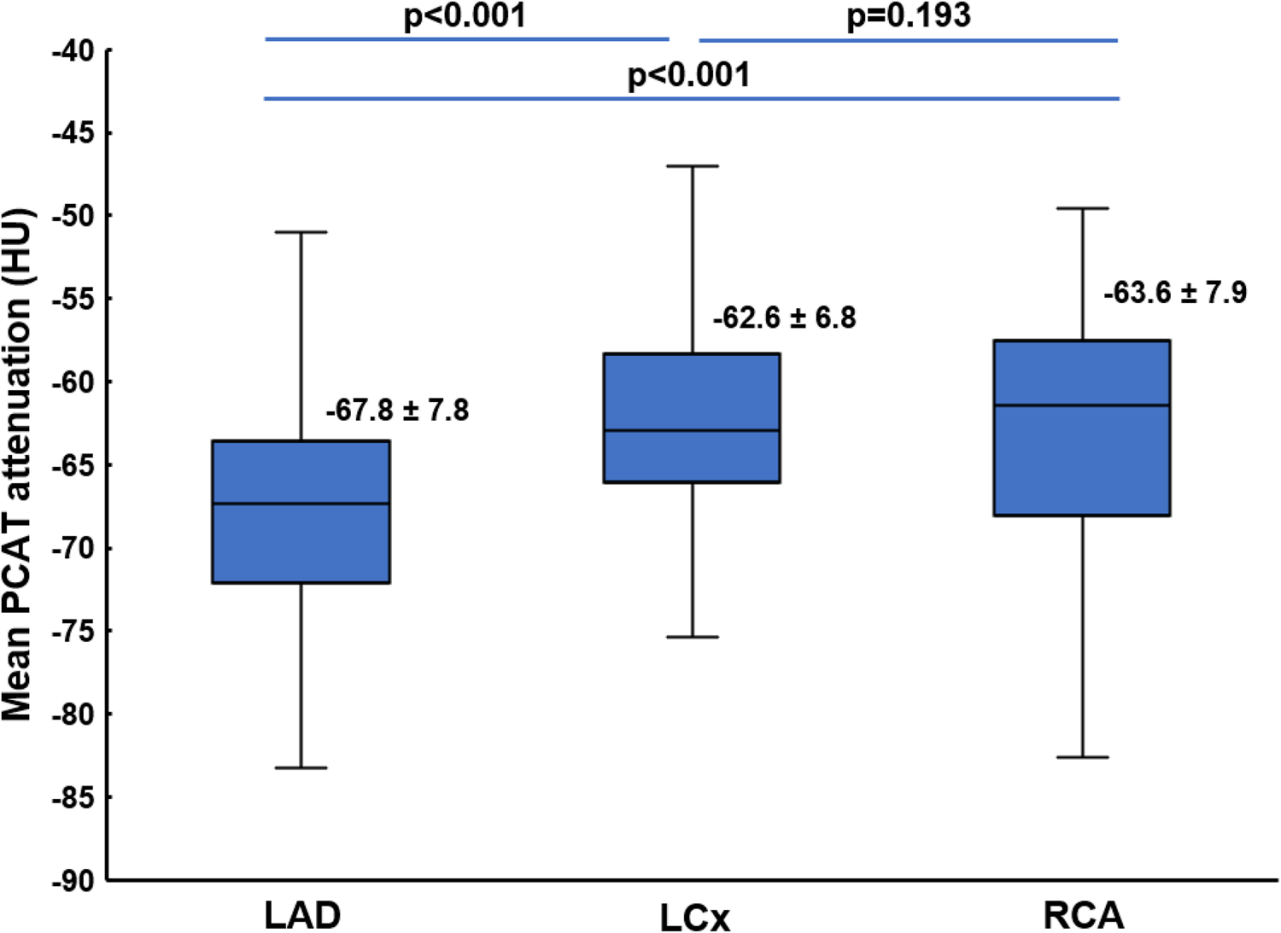
The comparison of the mean pericoronary adipose tissue (PCAT) attenuation among the left anterior descending artery (LAD), the left circumflex artery (LCx) and the right coronary artery (RCA)

The mean PCAT attenuation of the overall population was − 64.4 ± 8.0 HU. The distribution of the mean PCAT attenuation around the epicardial coronary arteries is shown in Fig. 3. The mean PCAT attenuation was significantly lower in the LAD vs. the LCx and vs. the RCA (− 67.8 ± 7.8 HU vs − 62.6 ± 6.8 HU vs − 63.6 ± 7.9 HU, respectively, p < 0.001, Fig. 4). In addition, no significant correlations existed between the three individual coronary arteries with regard to the mean PCAT attenuation (Supplemental Fig. 1).

### Sex differences in pericoronary adipose tissue

**Fig. 5:**
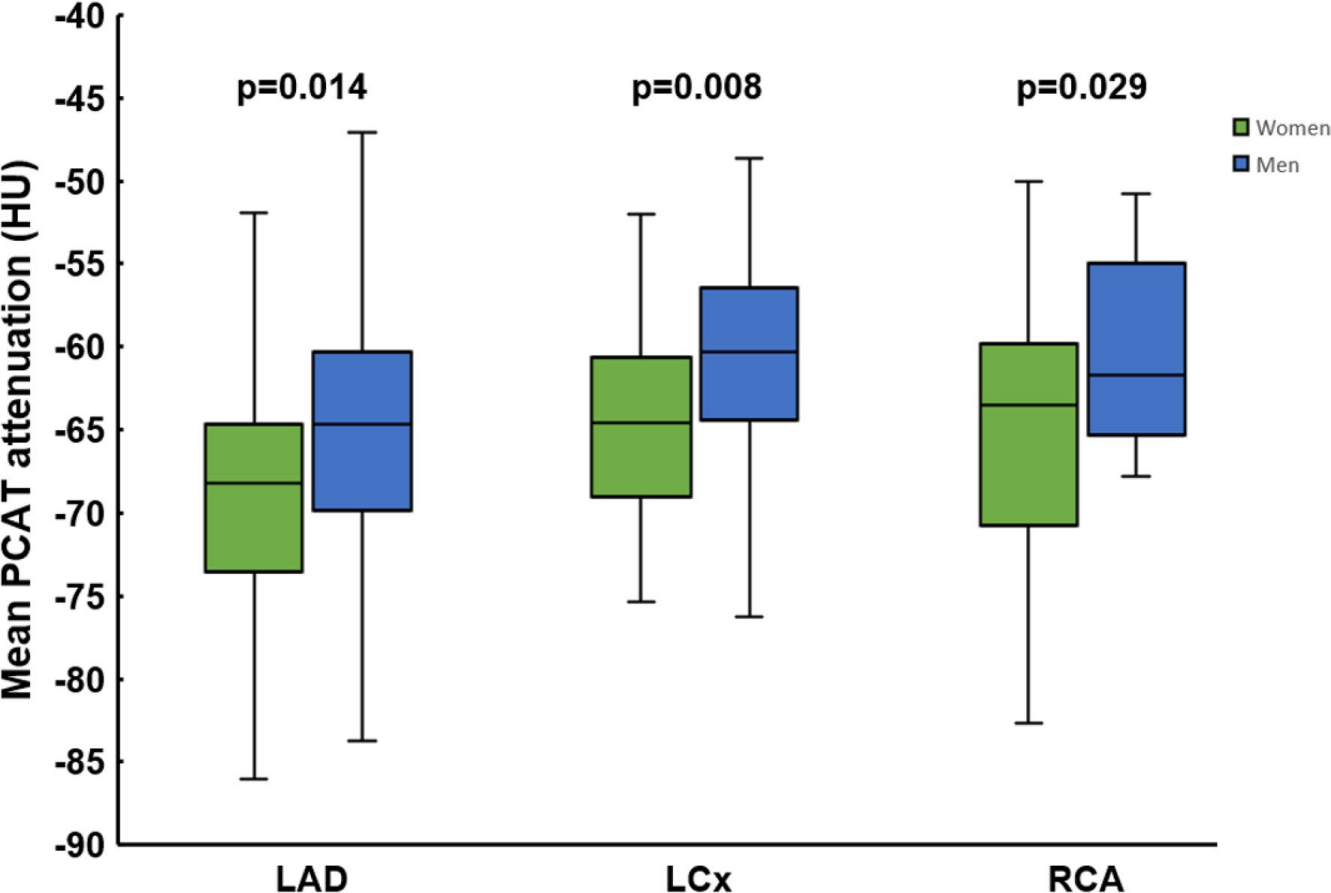
The mean pericoronary adipose tissue (PCAT) attenuation among the left anterior descending artery (LAD), the left circumflex artery (LCx) and the right coronary artery (RCA) according to sex

At a per-person level, the mean PCAT attenuation was significantly higher in men as compared to women (− 62.7 ± 7.9 HU vs − 66.3 ± 7.5 HU, p < 0.001). Moreover, this sex-related difference in mean PCAT attenuation was noted in each of the three epicardial coronary arteries (LAD: − 65.7 ± 7.6 HU vs − 69.4 ± 7.6 HU, p = 0.014; LCx: − 60.6 ± 7.4 HU vs − 64.3 ± 5.9 HU, p = 0.008; RCA: − 61.7 ± 7.9 HU vs − 65.0 ± 7.7 HU, p = 0.029, respectively, Fig. 5). Sex remained independently associated with the mean PCAT attenuation after adjustment for smoking status (β coefficient: 3.3 (95% Confidence Interval: 1.56; 5.07, p < 0.001).

## Discussion

The current study assessed the mean PCAT attention in individuals without coronary atherosclerosis. The results demonstrate that the mean PCAT attenuation is significantly different between the LAD and RCA, and between the LAD and LCx. In addition, mean PCAT attenuation values were significantly higher in men compared to women in all three coronary arteries.

Previous studies have shown that many coronary artery plaque ruptures arise from non-obstructive atherosclerotic lesions [13]. Consequently, early identification of potentially vulnerable atherosclerotic lesions becomes increasingly relevant. Detection and quantification of vascular inflammation may further improve early risk stratification of patients, possibly even before the development of significant coronary artery plaques. Previous studies have shown the feasibility of non-invasive assessment of PCAT attenuation with CCTA for the detection of vascular inflammation [4–6]. Specifically, significant differences in PCAT attenuation have been shown between diseased and non-diseased coronary arteries [14]. Moreover, increased PCAT attenuation has been demonstrated between culprit and non-culprit lesions in patients who subsequently developed an acute myocardial infarction [15]. In addition, PCAT attenuation was also increased in patients with flow-limiting coronary artery lesions as compared to patients with non-flow limiting lesions [5, 6].

Information regarding PCAT attenuation values in coronaries without atherosclerosis is lacking. A prior study evaluating mean PCAT attenuation values in patients without CAD, showed slightly lower values in the non-atherosclerotic coronary arteries, compared to coronary arteries with CAD [14].

### Differences in PCAT attenuation among the different coronary arteries

In the current study, significant differences in mean PCAT attenuation between the coronary arteries were observed. Mean PCAT attenuation around the proximal LAD was lower compared to the RCA and LCx. This could potentially be explained by differences in anatomy between the three coronary arteries. Furthermore, studies showed that among the three coronary arteries, the LAD is predominantly and earlier subject to atherosclerosis [16–19]. In addition, higher plaque and calcium deposit burden were observed in the LAD compared to the RCA and LCx [20–22]. The lower PCAT attenuation values in the LAD from our study, may suggest that PCAT attenuation could be linked to vessel vulnerability for CAD.

Ma et al. [14] analyzed all three coronary arteries and found lower PCAT attenuation values in the LAD as well. In addition, Gaibazzi et al. [23] showed significant differences between the LAD/RCA and the LCx in vessels with no or < 50% coronary artery stenosis at CCTA. The CRISP-CT study that incorporated PCAT attenuation in calculating the fat attenuation index (FAI) using a proprietary algorithm (CaRiHEART, Carito Diagnostics, Oxford, United Kingdom), showed no difference in perivascular FAI values between the three coronary arteries in patients with suspected CAD, but observed a difference in prognostic value between the three coronaries [7].

Previous studies mainly focused on the RCA to represent overall pericoronary attenuation, without evaluating potential differences between the RCA, LAD and LCx [6, 15, 24–26]. The proximal RCA is characterized by the absence of confounding non-fatty structures such as side branches, coronary veins, or myocardium, and also by the highest volume of surrounding adipose tissue [4, 27]. However, the current findings suggest that the mean PCAT attenuation measurement of the RCA is not interchangeable with the other coronary arteries.

### Sex differences in PCAT attenuation

In the current study a significant difference in PCAT attenuation was noted between men and women. This observation is in agreement with results published recently by Ma et al. [14] and Tzolos et al [28], showing significantly increased PCAT attenuation values in men versus women. Men are known to have an increased risk of developing CAD compared to women, and at a younger age [29, 30]. Increased PCAT attenuation in men might reflect an increased burden of coronary artery inflammation, that contributes to the progression of coronary atherosclerosis. Additionally, sex-specific hormones may further contribute to the increased PCAT attenuation in men [31]. Notably, the PCAT values obtained in present study are higher than those reported in previous studies. PCAT is relatively novel and many factors may influence this parameter. Considering technical factors, van Diemen et al. [32] showed significant differences in mean PCAT attenuation based on the CT scanner type used. Mean PCAT attenuation values using 64- and 256-slice CT scanners were − 72.2 HU and − 80.2 HU, respectively. Another key factor affecting the absolute PCAT attenuation is the kV setting and should be taken into account when evaluating PCAT attenuation. A higher tube voltage is associated with higher PCAT values [14]. Using different tube voltages necessitates adjustment for differences in PCAT attenuation as previously validated [12], but this is only done by a limited amount of studies [7, 33]. Furthermore, PCAT attenuation is quantified in different centres by different software packages.

We think that absolute values of PCAT attenuation on CCTA need to be tested and validated across different CT scanners, tube voltages and software packages in different centres before standardized thresholds for PCAT attenuation can be defined and clinical application is possible.

### Study limitations

This is a single-centre, retrospective observational study with a limited patient cohort. The observational design of the study has inherent limitations including selection bias and unmeasured confounding. Furthermore, this study could not characterize the patients regarding their ethnicity and explore ethnic differences in PCAT attenuation.

## Conclusions

PCAT attenuation values were derived from CCTA images of coronary arteries without atherosclerosis. Mean PCAT attenuation differed significantly between the three coronary arteries and mean PCAT attenuation was significantly higher in men compared to women.

## Supplementary Information

Below is the link to the electronic supplementary material.

Supplementary file1 (DOCX 115 kb)

## Abbreviations

CAD: Coronary artery disease
CCTA: Coronary computed tomography angiography
LAD: Left anterior descending artery
LCx: Left circumflex artery
LM: Left main artery
PCAT: Pericoronary adipose tissue
RCA: Right coronary artery
