# Supplementary material for: Vessel and sex differences in pericoronary adipose tissue attenuation obtained with coronary CT in individuals without coronary atherosclerosis
Source: Int J Cardiovasc Imaging. 2022 Aug 24;38(12):2781–9. doi: 10.1007/s10554-022-02716-7 (PMC9708810; doi:10.1007/s10554-022-02716-7)
Supplement: Supplementary file 1 — Supplementary file1 (DOCX 115 kb) [file 10554_2022_2716_MOESM1_ESM.docx]

**Supplemental Figure 1. Correlation of the mean pericoronary adipose tissue (PCAT) attenuation among the three epicardial coronary arteries.**

**
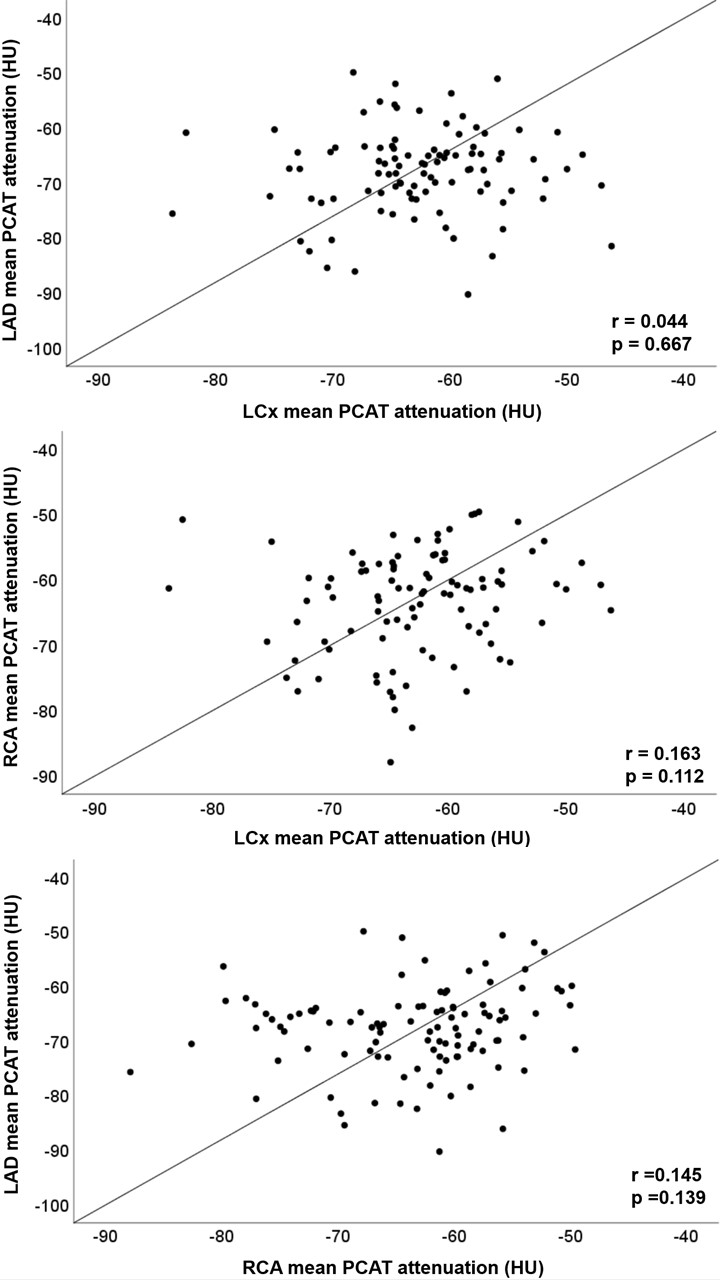
**
